# Partial replacement of soybean with alternative protein sources: Effects on meat quality, sensory attributes, and fatty acids and amino acids content of breast meat of a local chicken strain

**DOI:** 10.1111/jpn.14035

**Published:** 2024-08-12

**Authors:** Servet Yalçin, Sezen Özkan, Muazzez Cömert Acar

**Affiliations:** ^1^ Department of Animal Science, Faculty of Agriculture Ege University İzmir Turkey

**Keywords:** agri‐industrial byproducts, alternative feedstuffs, black soldier larvae meal, chicken breast meat, soybean

## Abstract

The environmental sustainability of soybean cultivation has been questioned as it has been linked to deforestation, eutrophication, pesticide use, and carbon dioxide footprint. Agri‐industrial byproducts and black soldier fly (BSF) larvae meal are promising alternative protein sources that can be used to partly replace soybean in broiler diets. The present study aimed to investigate the effect of partial replacement of soybeans with agro‐industrial by‐products with or without the addition of BSF dried larvae meal on the meat quality, fatty acid and amino acid content, and sensory traits of breast meat of local chickens. A total of 252 one‐day‐old mixed‐sex chicks from the Anadolu‐T pure dam line were randomly assigned to 1 of 3 diets; a soybean‐based Control diet, a diet in which soybean meal was partly replaced (SPR) with brewers' dried grain, sunflower seed meal, and wheat middlings and an SPR + BSF diet in which 5% of BSF dried larva meal was added to the SPR diet. All birds were slaughtered at a commercial slaughterhouse at 55 days and breast and drumstick muscles were sampled for meat quality analysis from 18 chickens/dietary treatments. No significant effects of diets were observed for the pH_24_ and lightness, redness, and yellowness of breast and leg meats. Thawing loss significantly decreased and cooking loss increased in the breast meat of chickens fed the SPR + BSF compared with those fed Control and SPR diets. Diets did not affect either texture profile or consumer sensory properties of breast meat. The chickens fed the SPR + BSF had the highest total saturated fatty acid and lower polyunsaturated fatty acid content in breast meat than those fed the Control and SPR diets. The essential and nonessential amino acid content of breast meat decreased by the SPR diet compared with the SPR + BSF diet. The chickens fed SPR + BSF diet had higher values of tasty, aromatic, and umami‐related amino acids than those fed SPR and Control diets. In conclusion, the results of the present study indicated that agri‐industrial byproducts with or without BSF larvae meal could be used to partially replace soybean meal in broiler diets without affecting technological meat quality traits. The addition of BSF larvae meal to the diet along with agri‐industrial by‐products improved the amino acid content of the breast meat of chickens but reduced polyunsaturated fatty acid levels.

## 1 INTRODUCTION

Worldwide meat production has been primarily driven by poultry meat in many regions of the world. While global sustainability in food production is increasingly important, chicken meat has a relatively low environmental impact compared with red meat (Tetteh et al., 2022; Williams et al., 2006). However, the cultivation of feedstuffs, processing, and transportation remain environmental challenges to be overcome in poultry meat farming due to global warming, energy use, and land use (Leinonen et al., 2012). Soybean is the main protein source in broiler diets and is mainly linked to the environmental impact of chicken meat production. In addition, the dependency on protein sources imported from overseas and the increase in soybean prices (Research and Markets, 2021) necessitates the search for alternative raw materials, such as regionally produced agri‐industrial byproducts (Georganas et al., 2023).

Sunflower meal, dried brewer grain, and wheat middlings, which are agricultural and industrial by‐products, can be used to partly replace soybean in chicken diets without negatively affecting performance (Denstadli et al., 2010; Jaroni et al., 1999; Rama Rao et al., 2006; Mbukwane et al., 2022). However, there are a limited number of studies on the effect of agri‐industrial by‐products used as an alternative feedstuff on chicken meat quality. Yaqoob et al. (2022) reported that the pH_24_ of breast and leg meat and the cooking loss of leg meat decreased by increasing sunflower seed meal from 3% to 9% in broiler diets. The addition of 100 g/kg inclusion of brewers' dried grain to broiler diets did not affect pH_24_, water holding capacity, and a* and b* values but reduced the L* value (Parpinelli et al., 2020; Pires Filho et al., 2021). Ahmadi and Karimov (2010) observed that 30% of wheat middlings did not affect the yield of thigh and breast of broilers.

In recent years, there has been an increasing interest in the use of insects as an alternative protein source in poultry diets. *Hermetica illucens* (black soldier fly, BSF) larvae become an attractive protein source in chicken diets due to their excellent energy and digestible amino acid content and the eco‐friendliness of their production (Barragan‐Fonseca et al., 2017; De Marco et al., 2015; Schiavone et al., 2017). Several studies have shown that from 5% to 15% of BSF larvae defatted meal or 20% full‐fat BSF larvae meal increased body weight and improved the feed conversion ratio of broilers (Dabbou et al., 2018; de Souza Vilela et al., 2021; Schiavone et al., 2019). Total replacement of soybean oil with BSF larvae fat in broiler diets or partial replacement of soybean up to 15% defatted BSF larva meal in broilers and broiler quail diets did not influence breast and leg meat quality parameters, while it increased saturated fatty acid content (Cullere et al., 2018; Schiavone et al., 2017, 2019). In contrast, a decrease in pH and a lighter colour was found by receiving 5% partially defatted or full‐fat BSF larvae meal inclusion by Popova et al. (2020).

Over the last two decades, interest in local and slow‐growing chickens adapted to local environmental conditions has increased due to their fewer welfare problems (Cartoni Mancinelli et al., 2020; Cassandro et al., 2015). Besides, small and medium producers have a greater interest in local and slow‐growing chickens. In this context, the breeding and selection programme of broiler lines registered as “Anadolu‐T” has been continuing in Turkey (Erensoy & Sarıca, 2023). We have already reported that partial substitution of soybean with agri‐industrial byproducts (sunflower meal, brewers dried grain and wheat middlings) with or without the addition of 5% BSF larvae meal did not affect the growth performance of local slow‐growing and commercial broiler chickens (Acar et al., 2024). Since these diets do not affect growth performance, it is important to determine their effect on chicken meat quality traits. Therefore, the objective of the present study was to examine the effect of a diet in which soybean was partially replaced with agri‐industrial by‐products (SPR) and the addition of 5% BSF larvae to the SPR diet (SPR + BSF) on the meat quality, fatty acids and amino acids content, and sensory traits of breast meat of local slow‐growing chickens.

## 2 MATERIALS AND METHODS

The present study is a part of the ongoing PRIMA project “Alternative animal feeds in Mediterranean poultry breeds to obtain sustainable products” (SUSTAvianFEED) (Grant number 2015). All experimental procedures were approved by the Farm Animal Experiments Local Ethics Committee of the Faculty of Agriculture at Ege University (Approval no:2022/002,07.04.2022).

### 2.1 Animal material and diets

A detailed description of animal material and diets was previously reported (Acar et al., 2024). Briefly, a total of 252 one‐day‐old mixed‐sex chicks from the Anadolu‐T pure dam line (slow‐growing) were weighed, wing banded, and randomly distributed to 18‐floor pens in an environmentally controlled poultry house. The chicks were randomly assigned into 1 of 3 diets, each consisting of six replicate pens with 14 chicks (sex ratio was 6:8 or 8:6 in each pen) per replicate in a completely randomised design. The Control diet consisted of a corn‐soybean meal‐based diet. Two experimental diets were prepared by partly replacing soybean meal with brewers' dried grain, sunflower seed meal, and wheat middlings in the SPR diet and 5% of BSF dried larva meal was added to the SPR diet (SPR + BSF). Dried larvae meal was purchased from Germina Tarım Teknolojileri Tic. Şti. The diets were formulated to be isoenergetic and isonitrogenous. The composition and nutrient properties of the diets during the starter (0–10 days), grower (11–25 days), and finisher (26–55 days) periods are shown in Table 1. The fatty acids and amino acids content of sunflower meal, brewers' dried grain, wheat middlings, and BSF larvae are shown in Tables 2, and 3 respectively. The feed and water were provided ad libitum. The ambient temperature was initially 32°C and was gradually reduced to 24°C by Day 28 and maintained at this temperature until the end of the experiment. The lighting programme was 23 L:1D from Day 1 to 3 then gradually reduced to 18 L: 6D by 7 days and kept till the end of the experiment.

**Table 1:** Ingredients and chemical composition of the starter (0–10 days), grower (11–25 days) and finisher (26 to slaughter age) diets.

	Diets^a^								
	Starter			Grower			Finisher		
Feed ingredients	Control	SPR	SPR + BSF	Control	SPR	SPR + BSF	Control	SPR	SPR + BSF
Corn	45.28	39.18	41.30	51.24	44.44	46.54	57.34	47.44	49.64
Wheat	11.86	12.50	12.18	14.86	14.50	14.50	15.00	15.50	15.50
Soybean meal	34.33	29.80	25.10	27.90	21.10	16.30	23.20	14.60	9.70
Sunflower meal	‐	3.58	3.63	‐	6.30	6.30	‐	8.00	8.00
Brewer's dried grain	‐	2.58	2.63	‐	3.08	3.08	‐	4.00	4.00
Wheat middlings	‐	2.58	2.63	‐	3.08	3.08	‐	4.00	4.00
BSF larvae	‐	‐	5.00	‐	‐	5.00	‐	‐	5.00
Sunflower oil	5.88	7.13	4.88	4.00	5.50	3.20	3.00	5.00	2.70
Limestone	0.50	0.50	0.50	0.30	0.30	0.30	0.20	0.20	0.20
DCP	1.00	1.00	1.00	0.80	0.80	0.80	0.60	0.60	0.60
Vitamin + mineral premix^b^	0.25	0.25	0.25	0.25	0.25	0.25	0.25	0.25	0.25
NaCl	0.20	0.20	0.20	0.20	0.20	0.20	0.20	0.20	0.20
Lysine (HCL ‐ % 78)	0.50	0.50	0.50	0.30	0.30	0.30	0.15	0.15	0.15
Methionine dl (mash ‐ % 99)	0.10	0.10	0.10	0.05	0.05	0.05	0.01	0.01	0.01
Threonine	0.05	0.05	0.05	0.05	0.05	0.05	‐	‐	‐
Rovabio + Natuphos E	0.05	0.05	0.05	0.05	0.05	0.05	0.05	0.05	0.05
Analysed nutrient composition									
ME kcal/kg	2984	2992	2991	2923	2921	2919	2904	2904	2903
CP, % diet	20.78	20.74	20.78	18.68	18.65	18.64	17.00	17.05	17.04
EE, % diet	8.49	9.41	9.38	6.63	8.11	7.72	5.79	7.52	7.52
CF, %diet	2.91	3.70	3.94	2.61	3.69	3.92	2.35	3.77	3.99
Ca, % diet	1.08	1.08	1.13	1.04	1.03	1.08	1.02	0.99	1.05
Total P, % diet	0.50	0.55	0.55	0.44	0.52	0.52	0.38	0.47	0.48

^a^ Diets: Control: soybean‐based diet; SPR: soybean in the Control diet was partially replaced by sunflower meal, brewer's dried grain, and wheat middlings; SPR + BSF: black soldier fly dried larvae were added to SPR.

^b^ Vitamin + mineral premix: Provided per 2.5 kg feed of diet: Vitamin A, 15,000,000 IU; vitamin D3, 3,000,000 IU; vitamin E, 50,000 mg; Vitamin K3, 4000 mg; vitamin B1, 3000 mg; Vitamin B2, 6000 mg; Niacinamid, 40,000 mg; Vitamin B6, 5000 mg; Vitamin B12, 30 mg; cCalcium‐d‐pantothenate 15,000 mg, Biotin, 75 mg; folic acid, 1,000 mg; choline chloride, 400,000 mg; manganese 80,000 mg; iron: 60,000 mg; copper, 5000 mg; zinc, 60,000 mg; iodine; 2000 mg; selenium 150 mg.

**Table 2:** Analysed fatty acid (% lipid) compositions of the sunflower meal, brewers dried grain, wheat middlings, and BSF larvae meal.

Fatty acids	Sunflower meal	Brewers dried grain	Wheat middlings	BSF larvae meal
Capric (C10:0)	0.497	0.365	0.000	0.785
Lauric (C12:0)	10.590	0.589	0.085	41.44
Myristic (C14:0)	3.588	0.664	0.000	9.173
Myristoleic (C14:1)	0.000	0.367	0.156	0.250
Pentadecanoic (C15:0)	0.000	0.252	0.000	0.106
Palmitic (C16:0)	10.176	22.947	17.811	17.58
Palmitoleic (C16:1)	2.234	0.284	0.421	2.382
Heptadecanoic (C17:0)	0.754	0.087	0.000	0.132
cis‐10‐heptadecanoic (C17:1)	0.000	0.000	0.000	0.222
Stearic (C18:0)	4.022	1.865	0.875	2.398
Elaidic (C18:1 trans)	0.720	0.000	0,000	0.329
Oleic (C18:1 cis	23.277	12.315	15.211	13.041
Linoleic (C18:2 cis)	37.469	51.271	56.912	9.708
Arachidic (C20:0)	0.5719	0.303	0.281	0.179
cis‐11‐eicosenoic A.(C20:1)	1.523	5.253	1.376	0.674
Linolenic A.(C18:3n3)	1.456	0.920	5.985	0.905
cis‐11.14‐ eicosadienoic (C20:2)	0.000	0.440	0.000	0.078
Behenic (C22:0)	0.594	0.305	0.000	0.062
Erucic A. (22.1)	0.000	0.177	0.557	0.017
cis‐11.14.17‐eicosatrienoic (C20:3n6)	0.000	0.045	0.000	0.000
Arachidonic (C20:4)	0.000	0.232	0.000	0.098
Tricosanoic (C23:0)	0.000	0.200	0.000	0.000
cis‐13.16‐docosadienoic (C22:2)	0.000	0.070	0.000	0.059
Lignoceric (C24:0)	0.000	0.219	0.000	0.018
cis‐5.8.11.14.17‐eicosapentaenoic (C20:5)	0.000	0.186	0.000	0.000
Nervonic (C24:1)	1.006	0.072	0.000	0.042
cis‐4.7.10.13.16.19‐docosahexaenoic (C22:6)	0.539	0.299	0.000	0.025
∑ Saturated	30.793	27.796	19.208	71.810
∑Monounsaturated	28.760	18.291	17.565	16.898
∑ Polyunsaturated	39.464	53.840	62.897	10.873
PUFA/SFA	1.281	1.936	3.275	0.151

Abbreviations: BSF, black soldier fly dried larvae; PUFA, polyunsaturated fatty acids; SFA, saturated fatty acids.

**Table 3:** Amino acids profile of sunflower meal, brewers dried grain, wheat middlings, and black soldier fly (BSF) larvae meal (% in each feedstuff).

Amino acids	Sunflower meal	Brewers dried grain	Wheat middling's	BSF larvae
Essential amino acids (EAA)				
Lysine	1.205	0.855	0.708	1.290
Leucine	2.320	1.075	1.062	1.500
Isoleucine	2.820	0.855	0.566	0.850
Valine	2.005	1.430	0.797	1.520
Methionine	0.240	0.215	0.283	0.615
Threonine	1.770	1.055	0.655	2.620
Phenylalanine	1.440	0.995	0.690	0.625
Histidine	0.355	0.205	0.460	0.335
Tyrosine	0.445	0.380	0.460	0.730
Tyriptofan	0.010	0.010	0.230	0.050
∑EAA	12.610	7.075	5.911	10.135
Nonessential amino acids (NEAA)				
Aspartic acid	2.855	1.085	1,151	2.160
Glutamic acid	4.985	2.780	3.522	1.750
Serine	1.205	0.690	0.743	0.800
Glutamine	0.095	0.040	‐	‐
Alanine	1.565	1.055	0.708	1.820
Glycine	3.245	1.350	0.850	‐
Proline	0.695	0.035	1.115	0.525
Hydroxyproline	0.965	0.765	‐	0.825
∑NEAA	15.610	7.80	8.089	7.880
∑AA	28.220	14.875	14.000	18.015

Abbreviation: BSF, black soldier fly dried larvae.

On Day 55 of the experiment, after 8 h without feed, all chickens were individually weighed (average weight was 2247 g) and transported to a commercial poultry slaughterhouse. The birds were electrically stunned, bled, scalded at 58°C, de‐feathered, eviscerated, and chilled. The breast and legs were separated from the carcass. Breast and drumstick were sampled from randomly selected 18 carcass/dietary treatments, packed in polyethylene bags, stored at 4°C, and transported to the laboratory for meat quality measurements.

### 2.2 Meat quality analysis

The left breast muscle was used for meat quality analysis. The pH_24_ of chicken breast samples was measured at 24 h post‐mortem by using a portable pH metre (Vuille, LabSen 252). The colour profile representing lightness (L*), redness (a*), and yellowness (b*) was determined by the Minolta colorimeter (Minolta CR300; Konica Minolta). Chroma and Hue were calculated using the formulas Croma = √ (a*)^2^ + (b*)^2^) and Hue = arctangent (b*/a*) respectively. To determine drip loss, approximately 4 cm of breast meat sample was weighed and suspended on a hook in individually packaged plastic bags. The samples were reweighed after being stored at 4°C for 72 h and drip loss was calculated as a percentage of the initial sample weight. Approximately 60 g of breast samples were cooked in a water bath to an endpoint temperature of 75°C, cooled to room temperature, and reweighed to determine the cooking loss as a percentage of the initial raw sample weight. The pH_24_ and colour measurements were repeated in individual drumstick samples from each dietary group.

Texture profile analysis (TPA) was performed using a TA‐XT2 Texture Analyzer (Stable Micro Systems) equipped with a 50 kg load cell. The pre‐test speed was 1 mm/s, test, and post‐test speed was 5 mm/s. The 1 × 1 × 1 cm^3^ samples were compressed to 50% of their original height by using a cylinder probe (P/36 R). Force‐time curves were used to evaluate the texture of the samples in terms of hardness, springiness, cohesiveness, gumminess, and chewiness.

### 2.3 Fatty acids and amino acids profiles of breast meat

The right breast meat from individual birds/dietary treatments was used to analyse fatty acids and amino acid contents. The fatty acid composition was determined by gas‐chromatography after the lipid was extracted according to Folch et al. (1957). The analyses were performed on an Agilent 6890 Network Gas Chromatograph (CA 95051). The column was an Agilent HP‐88 capillary (100 m × 0.25 mm ID × 0.2 μm). The oven temperature was held at 120°C for 1 min then increased to 240°C at a rate of 4°C/min, and held for 5 min. Injector and detector temperatures were 250 and 260°C respectively. Total saturated (SFA), monounsaturated (MUFA), polyunsaturated (PUFA) fatty acids, nutritive value index (NVI = (C18:0 + C18:1n9)/C16:0), atherogenicity index (AI = C12:0 + (4 XC14:0) + C16:0)/(∑ UFA) were calculated (Chen et al., 2016; Ulbricht & Southgate, 1991).

Amino acid analysis of breast meat samples was assessed by high‐performance liquid chromatography (HPLC) (Agilent 1260 Infinity II). The method consists of hydrolysing 0.5 g of samples using 5 mL 6 N hydrochloric acid. A 250 μL of 2 mM phenol was added to prevent oxidation, and 2 mL of 2% DTDPA solution was added to optimise the recoverability of cysteine, methionine, and tyrosine. The samples were kept in an oven at 110°C for 24 h and pH was adjusted to near neutral (6.7–7.3). The samples were centrifuged for 5 min at 1968 x g, the supernatant was filtered and analysed using HPLC (Agilent 1260 Infinity II). Total essential (EAA) and nonessential (NEAA) amino acids, the ratios of EAA/total amino acids and EAA/NEAA and total aromatic amino acids (AAA = phenylalanine + tryptophan + tyrosine), tasty amino acids (tasty AA = asparagine + thereonine + serine + glutamic + glycine + alanine), aromatic amino acids (aromatic AA = phenyl alanine + trptophan + thyrosine) and flavour related amino acids (flavour related AA = valine + isoleucine + leucine + phenyl alanine + proline) were calculated (Ali et al., 2019; Lengkidworraphiphat et al., 2021).

### 2.4 Sensory analysis

Sensory traits were evaluated using a descriptive panel of six semi‐trained panellists with ages ranging from 26 to 59 years. A total of 12 breast meat samples obtained from each dietary treatment were kept at −20°C. The frozen samples were thawed overnight at 4°C and then cooked to an internal temperature of 70°C. The cooked breast samples were cut into small pieces of approximately 2 × 2 × 3 cm and served on plates to panellists with a three‐digit blind code. Water and crackers were available for panellists to remove the residual flavour from the previous sample. The breast samples were evaluated for appearance, juiciness, flavours, texture, and overall liking over two sessions on two consecutive days. The five categorical scales (1 = do not like, 5 = extremely liked) were used to indicate liking for all parameters (Semjon et al., 2020).

### 2.5 Statistical analysis

The data were analysed by one‐way analysis of variance (ANOVA) where the diets were fixed main effects using JMP software (JMP, Pro 17, SAS Institute). The experimental unit was an individual bird/dietary treatment to analyse meat quality and profiles of fatty acids and amino acids. Data were tested for normality before ANOVA analysis using the Kolmogorov–Smirnov test. The significant differences among dietary groups were tested using the Student‐*t* test.

Sensory attributes of the appearance, juiciness, flavours, texture, and overall liking of breast meat were evaluated by chi‐square. Principle component analysis (PCA) was also used for various variables to describe the sensory quality of meat samples. A dataset consisting of consumer sensory attributes in each breast meat sample from each dietary treatment and evaluations of six panellists on two consecutive days was considered for PCA. Statistical significance was determined at *p* < 0.05.

## 3 RESULTS

No significant effects of diets were observed for the pH_24_ and colour of breast and leg meats (*p* > 0.05) (Table 4). While thawing loss significantly decreased (*p* = 0.001) and cooking loss increased (*p* < 0.001) in the breast meat of broilers fed SPR + BSF than those fed the Control and SPR diets, the lowest cooking loss was obtained in breast meat of chickens fed the SPR diet (Table 4). A higher chroma index (*p* = 0.018) was obtained in the breast meat of chickens fed SPR and SPR + BSF diets compared with those fed the Control diet. The diets did not affect the hue index of breast meat and chroma and hue indexes of leg meat. The texture profile of breast meat including hardness, springiness, gumminess, and chewiness was not affected by the diets (*p* > 0.05). SPR and SPR + BSF diets tended to decrease cohesiveness in the breast meat (*p* = 0.066). Springiness ranged between 0.064 and 0.083 while gumminess ranged between 2.48 and 3.32. The numerically lowest chewiness was for the breast meat from the SPR + BSF diet, however, it was not statistically significant (*p* = 0.099).

**Table 4:** Quality traits of the breast and thigh meats by diets.

	Diets^1^				
Quality traits	Control	SPR	SPR + BSF	SEM^2^	*p*‐Value
Breast		
Quality characteristics					
pH_24_	5.68	5.60	5.62	0.034	0.222
L*	55.11	54.29	54.16	0.754	0.451
a*	2.09	3.28	3.36	0.406	0.062
b*	3.00	3.76	3.63	0.341	0.267
Chroma index	33.65^b^	59.12^a^	58.58^a^	7.004	0.018
Hue	0.949	0.861	0.842	0.088	0.655
Drip loss	3.68	3.90	4.11	0.354	0.701
Thawing loss	6.98^a^	8.22^a^	5.39^b^	0.505	0.001
Cooking loss	17.98^b^	16.55^c^	19.42^a^	0.495	<0.001
Texture profile					
Hardness (N)	31.93	39.29	32.10	2.505	0.080
Cohesiveness	0.107	0.088	0.079	0.008	0.066
Springiness (mm)	0.083	0.074	0.064	0.006	0.133
Gumminess (N)	3.32	3.33	2.48	0.323	0.102
Chewiness (N*mm)	0.287	0.219	0.153	0.041	0.099
Leg					
Quality characteristics					
pH	6.39	6.30	6.23	0.053	0.132
L*	56.25	57.61	56.79	0.421	0.077
a*	3.10	3.23	2.92	0.218	0.647
b*	0.969	−0.0593	0.960	0.433	0.164
Chroma index	26.62	27.25	25.06	3.102	0.866
Hue	0.340	−0.014	0.329	0.119	0.071

*Note*: ^a,b^Means in the same row with different superscripts differ significantly (*p* < 0.05).

^1^ Diets: Control, soybean‐based diet; SPR, soybean in the Control diet was partially replaced by sunflower meal, brewer's dried grain, and wheat middlings; SPR + BSF, black soldier fly dried larvae were added to SPR.

^2^ SEM: Pooled standard error of the mean.

The LSMeans of the fatty acid profile of the breast meat samples by diets are presented in Table 5. The 16:0, 18:1 cis and 18:2 cis were the main fatty acids within total SFA, MUFA, and PUFA respectively. The 18:3 n‐3 and 21:0 content of breast meat were not influenced by diets (*p* > 0.05). The highest 18:1 cis (*p* < 0.001) and 20:3 n3 (*p* < 0.001) values were for the breasts from the Control diet. Both the SPR and SPR + BSF diets increased the 12:0 (*p* < 0.001) and 14:0 (*p* < 0.001) content of the breast meat compared with the Control while the meat samples from SPR + BSF had the highest values. The 16:0 (*p* < 0.001) and 16:1 (*p* < 0.001), 18:0 (*p* = 0.006), 18:1 cis (*p* < 0.001) and 20:3 n3 (*p* < 0.001) fatty acids of the breast meat of the SPR chickens were lower than those from the Control. Meat samples from the SPR + BSF had the lowest 18:1 cis, 20:0, 22:0, and 20:3 n3 values (*p* < 0.001). Total SFA content was the highest for the breast meat of chickens fed by SPR + BSF (*p* < 0.001) (Table 5). The highest total MUFA was obtained in the breast meat of birds fed the Control diet (*p* < 0.001) whereas chickens fed SPR and SPR + BSF had similar MUFA content. The SPR + BSF diet decreased while SPR increased the total PUFA content of the breast meat compared with the Control (*p* < 0.001). The PUFA/SFA levels of the breast meat decreased with SPR + BSF but increased with SPR compared with the Control (*p* < 0.001). SPR + BSF diet resulted in the lowest nutritive value and highest atherogenicity index (*p* < 0.001) (Table 5).

**Table 5:** Fatty acid composition (% of lipid) of breast meat by diets.

	Diets^1^				
Fatty acids	Control	SPR	SPR + BSF	SEM^2^	*p*‐Value
Lauric, 12:0	0.050^c^	1.061^b^	3.717^a^	0.262	<0.001
Myristic, 14:0	0.422^c^	0.797^b^	1.837^a^	0.096	<0.001
Palmitic, 16:0	19.69^b^	18.73^c^	21.05^a^	0.112	<0.001
Palmitoleic, 16:1	4.40^b^	3.99^c^	4.79^a^	0.064	<0.001
Stearic, 18:0	5.483^a^	5.272^b^	5.37^a,b^	0.042	0.006
Oleic, 18:1 cis	40.83^a^	38.88^b^	37.56^c^	0.249	<0.001
Linoleic, 18:2cis	26.13^b^	28.91^a^	22.95^c^	0.287	<0.001
Linolenic, 18:3 n3	0.507	0.506	0.504	0.009	0.965
Arachidic 20:0	0.307^a^	0.295^a^	0.244^b^	0.007	<0.001
Cis‐11 Eicosenoic 20:1	0.35^b^	0.374^b^	0.414^a^	0.011	0.004
Heneicosanoic 21:0	0.158	0.149	0.130	0.011	0.182
Behenic, 22:0	0.163^a^	0.158^a^	0.027^b^	0.025	<0.001
Eicosatrienoic, 20:3n3	0.354^a^	0.248^b^	0.169^c^	0.009	<0.001
∑ Saturated (SFA)	26.44^b^	26.6^b^	33.00^a^	0.391	<0.001
∑Monounsaturated (MUFA)	45.89^a^	43.43^b^	43.10^b^	0.227	<0.001
∑ Polyunsaturated (PUFA)	27.66^b^	29.92^a^	23.89^c^	0.294	<0.001
PUFA/SFA	1.05^b^	1.13^a^	0.72^c^	0.023	<0.001
Nutritive value index^3^	0.283^a^	0.286^a^	0.259^b^	0.003	<0.001
Atherogenicity index^4^	0.292^b^	0.288^b^	0.479^a^	0.003	<0.001

*Note*: ^a,b^Means in the same row with different superscripts differ significantly (*p* < 0.05).

^1^ Diets: Control, soybean‐based diet; SPR, soybean in the Control diet was partially replaced by sunflower meal, brewer's dried grain, and wheat middlings; SPR + BSF, black soldier fly dried larvae were added to SPR.

^2^ SEM: Pooled standard error of the mean.

^3^ Nutritive value index = (C18:0) + C18:1 n‐9)/C16:0.

^4^ Atherogenicity index = (C12:0 + (4×C14:0) + C16:9)/∑ UFA.

The diets did not affect the EAA of valine and methionine or the NEAA of alanine and serine in breast meat (*p* > 0.05) (Table 6). While breast meat lysine (*p* = 0.002), leucine (*p* = 0.018), isoleucine (*p* = 0.025), phenylalanine (*p* = 0.001), aspartic acid (*p* < 0.001) and glycine (*p* = 0.016) levels decreased in chickens fed SPR compared with those fed Control and SPR + BSF diets, glutamine (*p* = 0.009) decreased and histidine (*p* < 0.001), tyrosine (*p* = 0.005) and glutamic acid (*p* = 0.001) increased in chickens fed SPR + BSF compared with those fed Control and SPR diets (Table 6). The chickens fed the Control diet had intermediate levels of threonine and hydroxyproline (*p* = 0.011 and 0.015 respectively). Lower total EAA and NEAA levels were obtained in the breast meat of chickens fed the SPR diet compared with those fed the SPR + BSF diet (*p* = 0.014 and <0.001 respectively). The diets did not affect the EAA/NEAA ratio of breast meat (*p* > 0.05). Tasty (*p* = 0.001), aromatic (*p* < 0.001) and umami‐related (*p* < 0.001) amino acids were higher in birds fed SPR + BSF than those fed SPR and Control diets. Diet SPR lowered flavour‐related amino acids compared with Control while chickens from Control and SPR + BSF diets had similar values (*p* = 0.018) (Table 6).

**Table 6:** Amino acid compositions of the breast meats of broilers by diet.

		Diets^1^				
Amino acids		Control	SPR	SPR + BSF	SEM^2^	*p*‐Value
Essential amino acids (EAA)	Lysine	14.8^a^	8.2^b^	14.8^a^	1.202	0.002
	Leucine	31.4^a^	18.4^b^	28.2^a^	2.812	0.018
	Isoleucine	14.4^a^	9.6^b^	16.4^a^	1.537	0.025
	Valine	17.6	16.2	21.0	2.633	0.440
	Methionine	2.4	2.0	1.6	0.413	0.424
	Threonine	14.8^a,b^	4.4^b^	21.8^a^	3.412	0.011
	Phenylalanine	12.4^a^	4.6^b^	11.8^a^	1.244	0.001
	Histidine	3.4^b^	8.4^b^	22.4^a^	2.617	<0.001
	∑EAA	111.2^a,b^	71.8^b^	138.0^a^	13.311	0.014
Nonessential amino acids (NEAA)	Serine	2.2	2.6	2.0	0.813	0.876
	Aspartic acid	28.6^a^	13.8^b^	35.0^a^	2.122	<0.001
	Glutamine	6.8^a^	4.8^a^	1.6^b^	0.911	0.009
	Glutamic	23.4^b^	6.0^c^	28.4^a^	2.125	0.001
	Alanine	15.2	20.6	21.4	2.087	0.114
	Glycine	12.4 ^a^	6.4^b^	12.4^a^	1.423	0.016
	Proline	32.4 ^a^	18.8^b^	16.0^b^	2.701	0.002
	Hydroxyproline	18.0 ^a,b^	10.4^b^	23.2^a^	2.557	0.015
	Tyrosine	17.2^b^	17.4^b^	23.8^a^	1.207	0.005
	∑NEAA	156.2^a^	100.8^b^	163.8^a^	7.652	<0.001
	∑ AA	267.4^a^	172.6^b^	301.8^a^	20.001	0.002
	EEA/∑ AA	0.413	0.411	0.449	0.019	0.329
	EAA/NEAA	0.706	0.711	0.829	0.060	0.306
	Aromatic AA^3^	32.6^b^	25.2^c^	38.8^a^	1.702	<0.001
	Flavour related AA^3^	108.2^a^	67.6^b^	93.4^a,b^	8.589	0.018
	Tasty AA^4^	32.6^b^	25.2^c^	38.8^a^	1.713	0.001
	Umami AA^5^	52.0^b^	19.8^c^	63.4^a^	3.532	<0.001

*Note*: ^a,b^Means in the same row with different superscripts differ significantly (*p* < 0.05).

^1^ Diets: Control, corn‐soybean based diet; SPR, soybean in the Control diet was partially replaced by sunflower meal, brewer's dried grain, and wheat middlings; SPR + BSF, black soldier fly dried larvae were added to SPR.

^2^ SEM: Pooled standard error of the mean.

^3^ Aromatic AA = Phenyl alanine + trptophan + thyrosine; ^3^Flavour related AA = valine + isoleucine + leucine + phenyl alanine + proline.

^4^ Tasty AA4 = asparagine + thereonine + serine + glutamic + glycine + alanine.

^5^ Umami AA5 = glutamic + aspartic.

**Figure 1:**
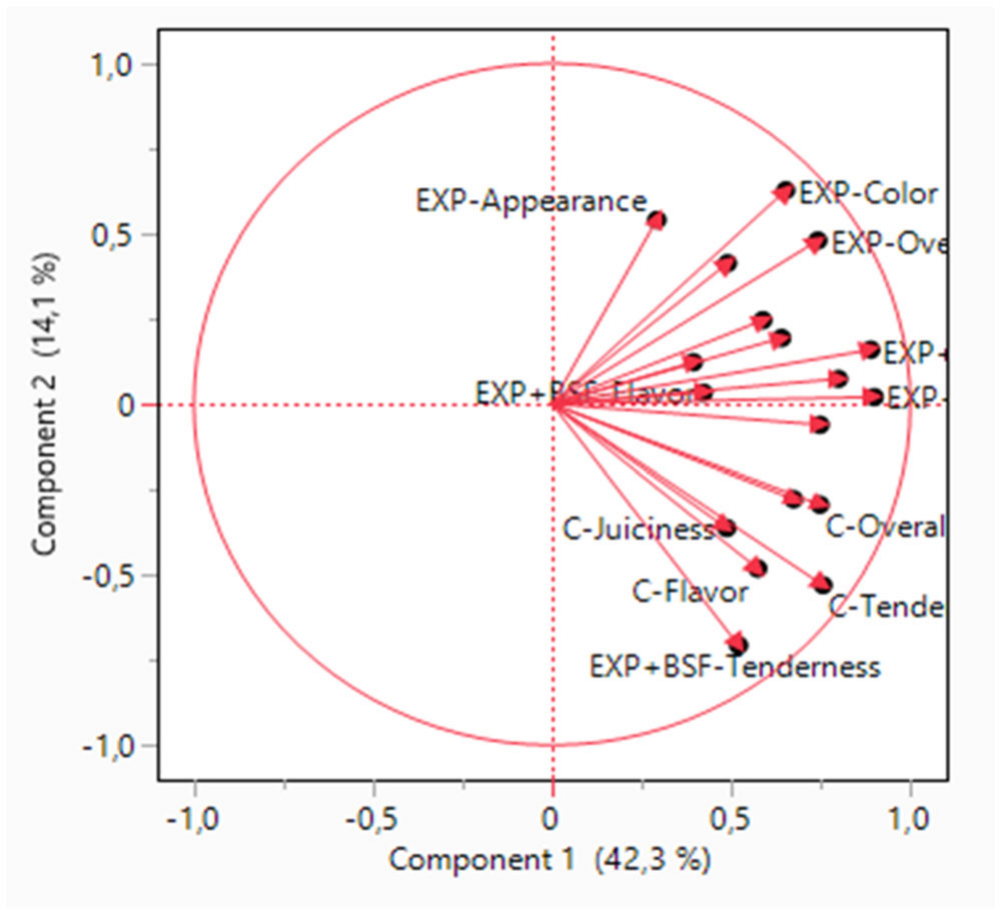
Principal component analysis (PCA) of sensory evaluation of breast meat samples of chickens. C: corn–soybean‐based diet, SPR: soybean in the Control diet was partially replaced by sunflower meal, brewer's dried grain, and wheat middlings; SPR + BSF: black soldier fly dried larvae were added to SPR.

Means (score) and frequency of consumer responses (%) of sensory evaluation of breast samples of chickens by diets are presented in Table 7. The diets did not influence consumer sensory traits of appearance, juiciness, flavours, texture and overall liking. The consumer score ranged between 3.8 and 4.4 for all traits. The PCA showed that about 85.31% of the total variation was explained by the first five components (Table 8). The loading plot provided a multidimensional visualisation of the effects of diets on consumer sensory scores (Figure 1) showing that the first two components explained 56.5% of total variation. The sensory parameters evaluated by panellists were close together.

**Table 7:** Means (score) and frequency of consumer responses (%) of sensory evaluation of breast samples of chickens by diets.

		Diets^a^		
Sensory parameters	Consumer sensory attributes	Control	SPR	SPR + BSF
Appearance				
*χ* ^2^ = 7.30 *p* = 0.294	Like extremely	50.0	50.0	50.0
	Like very much	33.3	8.3	41.7
	Like	16.7	33.3	8.3
	Dislike	0.0	8.3	0.0
	Dislike extremely	0.0	0.0	0.0
	Means	4.3	4.0	4.4
Colour				
*χ* ^2^ = 7.43, *p* = 0.283	Like extremely	58.3	41.7	41.7
	Like very much	33.3	25.0	50.0
	Like	8.3	25.0	0.0
	Dislike	0.0	8.3	8.3
	Dislike extremely	0.0	0.0	0.0
	Means	4.5	4.0	4.3
Flavour				
*χ* ^2^ = 4.54 *p* = 0.337	Like extremely	25.0	33.3	16.7
	Like very much	75.0	50.0	66.7
	Like	0.0	16.7	16.7
	Dislike	0.0	0.0	0.0
	Dislike extremely	0.0	0.0	0.0
	Means	4.3	4.2	4.0
Juiciness				
*χ* ^2^ = 3.61 *p* = 0.462	Like extremely	25.0	41.7	58.3
	Like very much	33.3	41.7	16.7
	Like	41.7	16.7	25.0
	Dislike	0.0	0.0	0.0
	Dislike extremely	0.0	0.0	0.0
	Means	3.8	4.3	4.3
Tenderness				
*χ* ^2^ = 1.411 *p* = 0.842	Like extremely	25.0	41.7	33.3
	Like very much	33.3	41.7	33.3
	Like	41.7	16.7	33.3
	Dislike	0.0	0.0	0.0
	Dislike extremely	0.0	0.0	0.0
	Means	3.8	4.3	4.0
Overall Liking				
*χ* ^2^ = 1.21 *p* = 0.877	Like extremely	16.7	25.0	33.3
	Like very much	66.7	58.3	58.3
	Like	16.7	16.7	8.3
	Dislike	0.0	0.0	0.0
	Dislike extremely	0.0	0.0	0.0
	Means	4.0	4.0	4.3

^a^ Diets: Control, soybean‐based diet; SPR, soybean in the Control diet was partially replaced by sunflower meal, brewer's dried grain, and wheat middlings; SPR + BSF, black soldier fly dried larvae were added to SPR.

**Table 8:** Principle component analysis results for the first five components.

Component	Eigenvalues	% of variance	Cumulative variance
1	7.61	42.29	42.29
2	2.54	14.13	56.42
3	2.16	12.00	68.43
4	1.76	9.76	78.19
5	1.28	7.12	85.31

## 4 DISCUSSION

The welfare of poultry production is becoming more important, which results in increased attention to local slow‐growing chickens. However, the environmental impact factor of chicken meat production using slow‐growing chickens is higher due to the later slaughter age and higher feed consumption compared with fast‐growing chickens (Dixon, 2020). Using alternative local feedstuffs by partially replacing soybeans may reduce the environmental impact of the production. Our results previously showed that a diet of soybean was partly substituted with local agri‐industrial by‐products and BSF meal did not affect performance and blood parameters of local slow‐growing chickens (Acar et al., 2024). The present study aimed to evaluate the meat quality of chickens fed by alternative protein sources.

Yaqoob et al. (2022) reported that when soybean‐based chicken diets were replaced with sunflower seed meal, pH_24_ of breast and leg meat and cooking loss of leg meat decreased by increasing sunflower meal from 3% to 9%. Ashour et al. (2019) reported that replacing soybean with 12% brewers dried grain increased L* and b*, decreased a*, and improved sensory properties. However, no studies have been found in which these two alternative protein sources were used together. There are contradictory results in the literature on the effect of BSF larvae meal on chicken meat quality depending on the larvae source and inclusion level. Cullere et al. (2016) found that the breast meat yield of quails was not influenced by substituting soybean with 10% and 15% of BSF larvae meal inclusion, while a reduced pH_24_ was obtained compared with the Control diet. They (Cullere et al., 2016) also noted that a* increased with 10% BSF larvae meal inclusion. Popova et al. (2020) stated that 5% of full‐fat BSF larvae meal inclusion decreased pH_24_ and increased L*, while not affecting a* and b*. In contrast, Pieterse et al. (2019) found no effect of the inclusion of dried BSF pre‐pupa up to %15 on pH, colour, thaw, and cooking loss of meat. However, no studies have been found in which agri‐industrial by‐products and BSF larvae meal are used together.

Our results showed that neither the SPR nor SPR + BSF diets affected the pH_24_ of breast and leg meat and the texture of breast meat which is important for meat quality, such as its juices, cooking loss, shelf life, and acceptability by consumers (Lyon & Lyon, 1997; Mir et al., 2017). A wide range of breast L* values have been reported due to slaughtering conditions, processing plants, and countries (Allen et al., 1998; Lee et al., 2022). The L* values range between 40 and 66 and can be classified as <48 dark, 48–56 normal, and >56 pale (Petracci et al., 2004; Qiao et al., 2001). The L* value in the present study was within the normal range and was not influenced by diets. There was no effect of diets on the a* and b* values of the breast and leg meats and was in the range reported by Petracci et al. (2004). Since the hue angle is determined by a* and b* and indicates colour intensity, the diet effect on hue angle was not significant, either. The highest chroma values obtained in breast meat from SPR and SPR + BSF compared with the Control diet explained the higher saturation of the hue (King et al., 2023). It is difficult to attribute this change in chroma to a single feed component, as soybean meals were partially replaced by sunflower meal, brewer's dried grain, and wheat middlings in the SPR diet and an additional 5% of BSF larvae meal in the SPR + BSF diet. However, previous results showed that soybean meal replacement with sunflower meal (from 0% to 9% in the diet) (Yaqoob et al., 2022) or BSF larva fat (from 0 to 90 g/kg in the diet) (Murawska et al., 2021) resulted in an increase in chroma in chicken breast (Kierończyk et al., 2023).

Drip loss, which indicates the water‐holding capacity of meat against gravity was not influenced by diets used in the present study. The partial replacement of soybean with sunflower meal, brewers' dried grain, and wheat middlings (SPR diet) decreased cooking loss compared with Control and SPR + BSF diets. This result indicated that the breast meat of chickens fed the SPR diet could retain moisture during cooking. The decrease in cooking loss in breast meat of broilers fed a diet of soybean partially replaced with sunflower meal (Yaqoob et al., 2022) or brewers' dried grain (Ashour et al., 2019) has previously been reported. While the incorporation of BSF larvae meal into the SPR diet increased cooking loss, it reduced thawing loss. Therefore, it can be concluded that both SPR and SPR + BSF affected water retention compared with the Control diet. It is known that pH influences water‐holding capacity (Qiao et al., 2001). In the present study, changes in pH after thawing were not measured. These changes in thawing loss and cooking loss may be related to the myofibrillar protein structure of the breast meat of chickens fed alternative diets, which should be investigated in the future (Zhang et al., 2022).

The fatty acid profile of the breast differed depending on the diet. Lauric and myristic acid content of BSF larvae meal was higher than those of sunflower meal, brewers dried grain, and wheat middlings. Therefore, the highest SFA levels in the breast of broilers fed the SPR + BSF diet were represented by lauric, myristic, and palmitic acids. Similarly, Schiavone et al. (2017) found a significant increase in lauric, myristic, and palmitic acid levels in the breast meat of chickens fed by a diet with partial or total replacement of soybean oil by BSF larvae fat. Due to the antimicrobial activity of lauric acid, which constitutes 11% of the SFA in breast meat chickens fed the SPR + BSF diet, might help to prevent antimicrobial resistance in humans (Borrelli et al., 2021). The increased saturated and monounsaturated fatty acids and decreased polyunsaturated fatty acids in the breast meat of chickens fed BSF larvae were reported by Kim et al. (2020). Our results showed that the SPR and SPR + BSF diets reduced MUFA content of the chicken breast meat; this was mainly due to lower oleic acid content in breast meat from SPR and SPR + BSF diets compared with the Control. The lower linoleic acid content of chicken meat from SPR + BSF diet compared with SPR resulted lower PUFA content. The PUFA/SFA index, ranging from 0.3 to 2.04 in chicken meat, is used to evaluate the effect of diet on cardiovascular health (Chen & Liu, 2020). In the present study, the SPR diet increased the PUFA/SFA ratio compared with the Control diet whereas SPR + BSF resulted in a lower PUFA/SFA ratio than the Control and SPR diets. The lowest PUFA/SFA level in the SPR + BSF breasts was predominantly due to the higher SFA levels. However, it was found still within the range in the literature. Ewald et al. (2020) reported that the fatty acid composition of larvae was affected by the nutritional composition of the insects' diet, larval weight, and age. Since a positive correlation between larvae weight and lauric acid and total SFA and a negative correlation with MUFA and PUFA content of larvae was reported (Ewald et al., 2020), studies examining different dietary combinations to balance fatty acid content of larvae are needed.

Straková et al. (2015) reported that glutamine, valine, isoleucine, leucine, phenylalanine, histidine, threonine, and alanine are the main amino acids in commercial chicken meat. The SPR diet reduced the total EAA and NEAA in the breast meat of chickens while chickens fed the SPR + BSF diet had similar amino acid levels to the Control. Our results reveal that the decrease in amino acid content in the breast meat of chickens fed the SPR diet was compensated by the inclusion of BSF larvae meal in the SPR diet. Humans can sense amino acids as bitter, umami, salty, sweet, or sour. Glutamic acid and aspartic acid are associated with the umami taste that enhances the flavour of chicken meat (Ali et al., 2019). Umami taste was significantly reduced by the SPR diet compared with the Control diet while adding BSF larvae helped to improve the test. Tasty and aromatic‐related amino acids were highest in the breast meat of chickens fed SPR + BSF compared with those fed Control and SPR diets. Similarly, Cullere et al. (2018) reported that when dietary protein sources were partly replaced with 10% and 15% defatted BSF larvae meal, most of the aromatic and tasty amino acids such as alanine, serine, aspartic acid, glutamic acid, tyrosine, and threonine were increased in breast meat of quails. However, in the present study, consumers were unable significantly to distinguish any differences among the breast meats of chickens fed different diets.

Cullere et al. (2019) found that replacing soybean oil with 50 or 100% BSF larvae fat as an alternative fat source had no significant effect on the sensory evaluation of the breast meat of chickens. However, Murawska et al. (2021) reported that a total replacement of soybean with full‐fat BSF larvae meal significantly lowered the taste intensity and juiciness of chicken meat. In the present study, no effects of diets on the consumer sensory scores were found, which may be due to the use of lower levels of BSF larvae meal compared with the cited literature. The results of PCA indicated that 85.3% of the total variance in the five considered variables such as appearance, juiciness, flavours, texture, and overall liking, were condensed into five new variables (PCs). The PCA failed to reveal clustering by diets and showed that sensory evaluation of the breast meat did not depend on the diets used in the present study. In the present study, the consumer sensory score test was designed with six panellists. It may be beneficial to repeat it with more panellists.

## 5 CONCLUSION

In conclusion, the results of the present study indicated that the alternative diets had no effect on pH_24_ and colour of chicken breast and leg meats. Although partial replacement of soybean with sunflower meal, brewers' dried grain, and wheat middlings reduced aromatic, flavour related, tasty, and umami amino acids, it did not affect the sensory and texture properties and nutritional value of chicken breast meat. The inclusion of 5% BSF larva meal to this diet improved amino acid content for aroma, flavour, tasty and umami flavours. However, BSF meal negatively affected the PUFA content and PUFA/SFA ratio and increased the atherogenicity index of the breast meat. Further research is needed to modulate the fatty acid profile of BSF larvae since published findings regarding the modulation of the fatty acid profile of BSF larvae appear promising (Rodrigues et al., 2022).

## CONFLICT OF INTEREST STATEMENT

The authors declare no conflict of interest.

## Data Availability

The datasets of the current study are not publicly available but are available from the corresponding author on request.
